# 630 nm light-emitting diode irradiation suppresses inflammation and promotes osteoblast differentiation in periodontal cell models – *in vitro* study

**DOI:** 10.1371/journal.pone.0351995

**Published:** 2026-10-22

**Authors:** Jin-Kyung Lee, Min-Seo Lee, Jae-Ho Jeong, Sun-Hee Ahn

**Affiliations:** 1 BioMedical Sciences Graduate Program (BMSGP), Chonnam National University, Hwasun, Jeollanam-do, Republic of Korea; 2 Department of Microbiology and Immunology, Chonnam National University Medical School, Hwasun, Jeollanam-do, Republic of Korea; 3 Bio & Health Photonics Research Center, Korea Photonics Technology Institute (KOPTI), Gwangju, Republic of Korea; Massachusetts General Hospital, UNITED STATES OF AMERICA

## Abstract

**Background:**

Periodontal disease involves persistent inflammation and alveolar bone loss. Photobiomodulation using light-emitting diodes (LEDs) may regulate inflammation and bone remodelling, but the optimal wavelength and irradiation timing remain unclear.

**Methods:**

Human gingival fibroblasts and RAW 264.7 macrophages were irradiated with 525, 630, or 850 nm LED light before or after lipopolysaccharide (LPS) stimulation. The effects of 630 nm irradiation were further evaluated in primary mouse calvarial osteoblasts, MC3T3-E1 cells, bone marrow-derived macrophages, and RAW 264.7 osteoclast precursors. Inflammatory mediators, nitric oxide production, cell viability, osteoblast differentiation, and osteoclast formation were assessed.

**Results:**

Among the wavelengths tested, 630 nm irradiation most consistently reduced pro-inflammatory cytokine expression and secretion and decreased nitric oxide production without affecting cell viability. In primary osteoblasts and MC3T3-E1 cells, 630 nm irradiation at 5 and 10 J/cm² increased alkaline phosphatase (ALP) activity, osteogenic marker expression, RUNX2 protein levels, and Smad1/5/9 phosphorylation. In contrast, the same irradiation conditions did not significantly affect osteoclast differentiation or the expression of osteoclast-related markers.

**Conclusion:**

Irradiation with 630 nm LED light suppressed inflammatory responses and promoted osteoblast differentiation without enhancing osteoclastogenesis. These findings suggest that 630 nm photobiomodulation may provide a balanced therapeutic approach for periodontal disease, although further validation in animal models and clinical studies is required.

## Introduction

Periodontal disease is one of the most prevalent chronic inflammatory disorders worldwide, affecting nearly half of adults over the age of 30 [1]. Clinically, it begins as gingivitis and may progress to periodontitis, with progressive destruction of the supporting structures of the tooth — the gingiva, periodontal ligament, cementum, and alveolar bone. The pathogenesis is now understood not simply as a bacterial infection but as a dysbiotic interaction between subgingival biofilms and a dysregulated host immune response [2,3]. Key periodontal pathogens such as *Porphyromonas gingivalis* and *Fusobacterium nucleatum* trigger pattern-recognition receptors on resident and infiltrating cells, leading to the production of pro-inflammatory cytokines, reactive oxygen species, and matrix-degrading enzymes [4,5].

Gingival fibroblasts are the most abundant cell type in the gingival connective tissue and act as a first-line sentinel population. When stimulated by bacterial lipopolysaccharide (LPS), they secrete IL-6 and IL-8, recruit immune cells, and produce matrix metalloproteinases that contribute to connective tissue breakdown [6]. Macrophages, in turn, orchestrate the subsequent phase of inflammation by coordinating immune-cell recruitment and regulating the balance between inflammatory amplification and resolution. Depending on the local microenvironment they polarize toward pro-inflammatory (M1) or pro-resolving (M2) phenotypes and produce mediators such as TNF-α, IL-1β, and nitric oxide (NO) [7]. Excessive or unresolved macrophage activation drives the chronicity of periodontal lesions and amplifies tissue damage.

Alveolar bone loss is the hallmark feature that distinguishes periodontitis from gingivitis. Persistent inflammation tips the balance of bone remodelling away from osteoblast-mediated formation and toward osteoclast-mediated resorption, partly through receptor activator of nuclear factor κB ligand (RANKL) upregulation and partly through direct cytokine effects on bone cells [8,9]. Therefore, any therapeutic strategy aimed at periodontitis must consider three interlinked targets: the inflammatory response of resident cells, the activity of bone-forming osteoblasts, and the activity of bone-resorbing osteoclasts.

Photobiomodulation (PBM) refers to the use of low-level red or near-infrared light, typically delivered by lasers or light-emitting diodes (LEDs), to modulate cellular activity in a non-thermal manner [10]. Mitochondrial chromophores, particularly cytochrome c oxidase (complex IV) in the mitochondrial respiratory chain, are widely regarded as primary photoacceptors of PBM. Photon absorption by this enzyme displaces inhibitory nitric oxide, increases mitochondrial membrane potential, and enhances ATP production [10,11]. However, photoreception in PBM is not restricted to mitochondrial chromophores. Additional photoresponsive targets have been proposed, including light-sensitive opsins (OPN2–OPN5), transient receptor potential (TRP) channels such as TRPV1, and other membrane photoacceptors that can modulate intracellular Ca² ⁺ signalling and downstream redox-sensitive pathways [12–14]. These non-mitochondrial mechanisms are increasingly recognised as contributors to the cellular effects of light, although current evidence indicates that opsin- and TRP-mediated responses are engaged mainly by blue and green wavelengths. Downstream, PBM has been reported to modulate reactive oxygen species (ROS) levels, activate nuclear factor kappa B (NF-κB) and mitogen-activated protein kinase (MAPK) signalling at low doses, and alter the expression of inflammatory cytokines [15]. Within periodontal research, several studies have examined laser- and LED-based PBM and reported anti-inflammatory effects in gingival cells [16], reductions in clinical indices of inflammation [17], and effects on osteoblast and osteoclast activity in isolated experiments [18,19].

Despite these advances, two practical questions remain underexplored. First, most published studies examine PBM either as a treatment applied after inflammation is established, or — less frequently — as a preventive measure applied before challenge. Direct comparisons of the two modes within the same experimental system are uncommon. Second, the inflammatory and bone-related effects of PBM are usually reported in separate studies using different irradiation parameters, which makes it difficult to identify a single set of conditions that benefits both axes simultaneously. To address these gaps, the present study evaluated the effects of a custom-built three-wavelength LED device (525, 630, and 850 nm) applied either before or after LPS stimulation on cytokine and NO responses in Human gingival fibroblasts (HGFs) and RAW 264.7 macrophages.

## Materials and Methods

### Ethics statement

All animal experiments were approved by the Institutional Animal Care and Use Committee (IACUC) of Chonnam National University (Approval No. CNU IACUC-YB-2019–50 and CNU IACUC-YB-2017–53), Gwangju, Republic of Korea.

### Animals

Wild-type C57BL/6J mice were purchased from Damul Science, Inc (Daejeon, Korea). Mice aged 8-week-old (body weight, 18-22g) were used in this study. Neonatal (3-day-old) C57BL/6J pups from the same colony were used for primary calvarial osteoblast isolation. The animals were housed under specific pathogen-free conditions at 22 ± 2 °C with a 12-h light/dark cycle and were provided food and water ad libitum. Animals were euthanized by cervical dislocation under isoflurane anesthesia prior to tissue collection, and all efforts were made to minimize suffering.

### Cell culture and reagents

Human gingival fibroblasts (HGFs) were obtained from the American Type Culture Collection (ATCC, Manassas, VA, USA). The murine macrophage line RAW 264.7 and the pre-osteoblast subclone MC3T3-E1 (Subclone 4) were purchased from the Korean Cell Line Bank (KCLB, Seoul, Korea) and from ATCC, respectively. All lines were authenticated by the suppliers and used within ten passages after receipt to minimize passage-associated phenotypic changes and experimental variability and to maintain consistency across experiments [20]. Cells were routinely screened for *Mycoplasma* contamination by PCR and were confirmed negative throughout the study. Human gingival fibroblasts (HGFs) and RAW 264.7 cells were maintained in Dulbecco’s Modified Eagle Medium (DMEM; Gibco, Grand Island, NY, USA) containing 10% heat-inactivated fetal bovine serum (FBS; Gibco) and 1% penicillin-streptomycin (GenDEPOT, Katy, TX, USA) [21,22]. MC3T3-E1 cells and mouse primary calvarial osteoblasts were maintained in alpha-modified Eagle’s medium (α-MEM; WELGENE, Gyeongsan, Korea) with 10% characterized fetal bovine serum (FBS; Hyclone, Logan, UT, USA) and 1% penicillin-streptomycin. All cultures were kept at 37 °C in a humidified atmosphere of 5% CO_2_. Lipopolysaccharide (LPS) from *Escherichia coli* O111:B4 (Sigma-Aldrich, St. Louis, MO, USA) was dissolved in sterile distilled water as a 5 µg/µL stock and diluted to 1 µg/mL in culture medium immediately before use.

### Light-emitting diode device and irradiation protocol

A custom three-wavelength LED module incorporated LEDs emitting at 525 nm (green; APHHS1005ZGCK, Kingbright), 630 nm (red; APHHS1005SURCK, Kingbright), and 850 nm (near-infrared; HIR16-213C/L423/TR8, Everlight Electronics Co., Ltd.). Each individual LED measured 1.0 × 0.5 mm, and 225 LEDs were arranged in a 15 × 15 matrix on a 5 × 5 cm board, giving a density of 9 LEDs/cm² (S1 Fig). An aluminium heat sink with eight fins (height approximately 3 cm) was attached to the rear of the board to dissipate heat during operation, and the surface temperature of the cell culture medium was monitored to ensure that it remained below 37.5 °C throughout each irradiation session. The optical output of the device was calibrated with a power meter (PM100D with S121C sensor head, Thorlabs, Newton, NJ, USA). The working distance between the LED surface and the bottom of the culture well was fixed at 1 cm to maintain a standardised non-contact irradiation geometry and ensure reproducible energy delivery, consistent with previously established *in vitro* LED irradiation protocols [22,23]. A schematic of the non-contact irradiation setup, including the LED module, culture plate, irradiation direction, and the 1 cm working distance, is shown in S2 Fig. The detailed light-emitting diode device and irradiation parameters are summarized in Table 1. In the inflammation experiments, HGFs were seeded in 24-well plates at 3.0 × 10⁴ cells/mL (1 mL/well) and RAW 264.7 cells at 8.0 × 10⁴ cells/mL (1 mL/well), and the plates were incubated overnight. The next day, LED irradiation was applied either 30 min before (“pre-irradiation”) or 30 min after (“post-irradiation”) the addition of 1 µg/mL LPS. Cells were then cultured for an additional 24 h. The energy density was kept constant at 10 J/cm² for all three wavelengths in the inflammation experiments. After 24 h, culture supernatants were collected for ELISA and the Griess assay, while adherent cells were used for RNA extraction and quantitative PCR. Osteoblast and osteoclast differentiation experiments were performed using 630 nm irradiation at energy densities of 0, 2.5, 5, and 10 J/cm². Irradiation was applied on days 0 and 4 during osteoblast differentiation and on days 0 and 2 during osteoclast differentiation. These schedules were selected to provide repeated light exposure within the respective differentiation periods, taking into account the longer course of osteoblast maturation and the relatively rapid early progression of RANKL-induced osteoclastogenesis [24,25].

**Table 1:** Light-emitting diode irradiation parameters.

*Parameter*	525 nm	630 nm	850 nm
Light source	LED	LED	LED
Spectral region	Green	Red	Near-infrared
Number and arrangement of LEDs	225, 15 x 15 array	225, 15 x 15 array	225, 15 x 15 array
LED dimensions	1.0 x 0.5 mm	1.0 x 0.5 mm	1.0 x 0.5 mm
LED density	9 LEDs/cm²	9 LEDs/cm²	9 LEDs/cm²
Irradiance mode	Continuous	Continuous	Continuous
Irradiance at the sample surface	16.6 mW/cm²	16.6 mW/cm²	16.6 mW/cm²
Radiant exposure	10 J/cm² (inflammation)	10 J/cm² (inflammation); 0/2.5/5/10 J/cm² (bone)	10 J/cm² (inflammation)
Exposure time for 10 J/cm²	10 min	10 min	10 min
Irradiation schedule	Inflammation: single 30 min pre-/post-LPS	Inflammation: single 30 min pre-/post-LPS; Osteoblast: days 0, 4; Osteoclast: days 0, 2	Inflammation: single 30 min pre-/post-LPS
Output measurement	Thorlabs PM100D + S121C power meter	Thorlabs PM100D + S121C power meter	Thorlabs PM100D + S121C power meter
Maximum culture-medium temperature	<37.5 °C	<37.5 °C	<37.5 °C

### Cell viability assay

Cell viability was assessed using the 3-(4,5-dimethylthiazol-2-yl)-2,5-diphenyltetrazolium bromide (MTT) assay, as described previously [26]. After the appropriate treatment, the medium was replaced with 0.5 mg/mL MTT solution (Sigma-Aldrich) and the plates were incubated for 3 h in the dark at 37 °C. The formazan crystals were dissolved in dimethyl sulfoxide (DMSO; Sigma-Aldrich) and absorbance was read at 540 nm on an Epoch microplate reader (BioTek, Winooski, VT, USA). Cell viability was expressed relative to the untreated control, which was set to 100%. Cell viability in the inflammation experiments was measured 24 h after irradiation. In the osteoblast differentiation, viability was assessed at days 1, 4, and 7. Osteoclast viability was assessed at day 4.

### Quantitative real-time PCR

Total RNA was extracted using QIAzol Lysis Reagent (Qiagen, Hilden, Germany) following the manufacturer’s protocol. The concentration and purity of the RNA were checked on a NanoDrop spectrophotometer (Thermo Fisher Scientific). One microgram of total RNA was reverse-transcribed using the PrimeScript RT Reagent Kit (Takara Biotechnology, Otsu, Japan). Quantitative PCR was carried out with Power SYBR Green PCR Master Mix (Applied Biosystems, Foster City, CA, USA) on a QuantStudio 3 system (Applied Biosystems). Each reaction was performed in technical triplicate from at least three independent biological replicates. Relative gene expression was calculated by the 2^−ΔΔCt^ method, as described previously [27], with GAPDH as the internal reference. Primer sequences are listed in Table 2.

**Table 2:** List of primers used in this study.

*Gene*	Forward sequence (5’-3’)	Reverse sequence (5’-3’)
*hGAPDH*	ACCCCTTCATTGACCTCAAC	CTTGACGGTGCCATGGAATT
*hIL-6*	AGGGCTCTTCGGGAAATGTA	TGCCCAGTGGACAGGTTTC
*hIL-8*	TTTCTGTTAAATCTGGCAACCCT	ATAAAGGAGAAACCAAGGCACAGT
*mGAPDH*	TGTGTCCGTCGTGGATCTGA	GATGCCTGCTTCACCACCTT
*mIL-6*	CTCGGCAAACCTAGTGAGTT	ACACACGGATATATTTTCTGACCACAGT
*mIL-8*	ACTGAGAGTGATTGAGAGTGGAC	AACCCTCTGCACCCAGTTTTC
*mIL-1β*	TTGACAGTGATGAGAATGACC	GCAGGTTATCATCATCATCC
*mALP*	TGGCCTGGATCTCATCAGTATTT	AGTTCAGTGCGGTTCCAGACA
*mOSX*	GGTCCCCAGTCGAGGAT	CTAGAGCCGCCAAATTTGCT
*mBSP*	CACCGCCCGAAGCCTAT	ATTCGTTGCCTGTTTGTTCGT
*mOPN*	CAAGCAGACACTTTCACTCCAATC	CCTCAGTCCATAAGCCAAGCTATC
*mCOL1A1*	GAAACCCGAGGTATGCTTGATC	CACTCGCCCTCCCGTCTT
*mc-Fos*	CGAAGGGAACGGAATAAGATG	GCTGCCAAAATAAACTCCAG
*mNFATc1*	ACCACCTTTCCGCAACCA	GGTACTGGCTTCTCTTCCGTTTC
*mCtsK*	GGACGCAGCGATGCTAACTAA	CAGAGAGAAGGGAAGTAGAGTTGTCACT

### ELISA

Culture supernatants from LPS-stimulated HGFs and RAW 264.7 cells were assayed for human IL-6 or mouse IL-6 using DuoSet ELISA kits (R&D Systems, Minneapolis, MN, USA) according to the manufacturer’s instructions [26]. Optical density was read at 450 nm with wavelength correction at 540 nm on an Epoch microplate reader.

### Nitric Oxide (NO) measurement

Nitrite accumulation in RAW 264.7 culture supernatants was used as a surrogate for NO production. Samples were mixed 1:1 with Griess reagent (Promega, Madison, WI, USA) and absorbance was read at 520 nm. Nitrite concentrations were calculated from a sodium nitrite standard curve [28].

### Osteoblast differentiation

Mouse primary calvarial osteoblasts were isolated from 3-day-old C57BL/6J neonates by sequential collagenase digestion as described previously [15], with minor modifications. Briefly, calvariae were aseptically dissected, cleaned of adherent connective tissue, and digested with 1 mg/mL collagenase type II (Sigma-Aldrich) at 37 °C; fractions 2–4 of the digest were pooled and used as the primary osteoblast population. Primary osteoblasts and MC3T3-E1 cells were plated at 2 × 10⁴ cells/mL in 24-well plates. The following day, the medium was changed to osteogenic medium consisting of α-MEM supplemented with 10% characterized fetal bovine serum (chFBS), 1% penicillin-streptomycin, and 100 ng/mL recombinant human BMP-2 (Sino Biological, Wayne, PA, USA) [27]. Irradiation with the 630 nm LED was applied on days 0 and 4 at the indicated energy densities, and the medium was refreshed every two days. Irradiation was performed at the onset of differentiation (day 0) and once during the differentiation period (day 4), the latter representing an early differentiation stage preceding the day-7 ALP endpoint, consistent with the reported onset of ALP activity from approximately day 3 of osteoblast differentiation [29]. On day 7, cells were fixed in 70% ethanol for 1 h at room temperature and stained for ALP using the manufacturer’s protocol (Sigma-Aldrich). ALP enzymatic activity was quantified in parallel cultures using the TRAcP & ALP Assay Kit (Takara Biotechnology). Each assay was performed at least three times independently.

### Osteoclast differentiation

Bone marrow was flushed from the femurs and tibiae of 8-week-old C57BL/6J mice with α-MEM, and bone marrow-derived macrophages (BMMs) were obtained by incubating the cell suspension overnight, collecting the non-adherent fraction, and culturing it in α-MEM supplemented with 10% chFBS, 1% penicillin-streptomycin, and 30 ng/mL recombinant mouse M-CSF (PeproTech, Rocky Hill, NJ, USA) for three days. Adherent cells were then switched to osteoclastogenic medium containing 30 ng/mL M-CSF plus 50 ng/mL recombinant mouse RANKL (PeproTech) and cultured for up to three additional days, with fresh medium supplied daily. RAW 264.7 cells, used as an alternative osteoclast precursor model, were cultured for four days in α-MEM supplemented with 10% FBS, 1% penicillin-streptomycin, and 50 ng/mL RANKL, with medium changes every two days. As with the osteoblast assays, irradiation was applied at the onset of differentiation (day 0) and once during the shorter four-day differentiation period (day 2), targeting the active pre-osteoclast fusion stage during which mononuclear precursors fuse and multinucleated cells begin to appear [30]. At the end of the culture period, cells were fixed in 3.7% formaldehyde for 30 min and stained for tartrate-resistant acid phosphatase (TRAP) using a commercial kit (Sigma-Aldrich). TRAP-positive multinucleated cells containing five or more nuclei were counted as mature osteoclasts under a light microscope (DFC450 C, Leica Microsystems, Wetzlar, Germany). Each assay was repeated at least three times independently.

### Western blot analysis

Western blot analysis was performed as described previously, with minor modifications [27]. Mouse primary calvarial osteoblasts were plated in 6-well plates at 1.0 × 10^5^ cells/well and cultured in osteogenic medium with or without 630 nm LED irradiation (days 0 and 4, at the indicated energy densities) [31,32]. On days 7 and 14, cells were rinsed twice with cold PBS and lysed in RIPA buffer containing protease and phosphatase inhibitors. Protein concentration was measured with the BCA assay (Pierce, Rockford, IL, USA). Equal amounts of protein (20 µg/lane) were separated by SDS-PAGE and transferred to PVDF membranes. Membranes were blocked in TBS-T containing 5% skim milk for 1 h at room temperature and incubated overnight at 4 °C with primary antibodies against RUNX2, phospho-Smad1/5/9, total Smad1 (all Cell Signalling Technology, Danvers, MA, USA), or β-actin (Sigma-Aldrich). After three washes with TBS-T, membranes were incubated with HRP-conjugated secondary antibodies for 1 h, washed again, and developed with Amersham ECL detection reagent. Images were captured on an EZ-Capture MG system (ATTO, Tokyo, Japan), and band intensities were quantified with ImageJ (version 1.53; NIH, Bethesda, MD, USA), normalized to β-actin (for RUNX2) or total Smad1 (for phospho-Smad1/5/9).

### Statistical analysis

All experiments were performed independently at least three times unless otherwise indicated. Quantitative data are expressed as mean ± standard deviation (SD). Normality of the data was assessed by the Shapiro-Wilk test. Comparisons between two groups were made using two-tailed Student’s *t*-test. Comparisons involving three or more groups were performed by one-way analysis of variance (ANOVA) followed by Dunnett’s *post hoc* test when each treatment group was compared with a single control, or by Tukey’s *post hoc* test for pairwise comparisons among all groups. A *p* value less than 0.05 was considered statistically significant. Statistical analyses and graph generation were performed in GraphPad Prism (version 9.0; GraphPad Software, San Diego, CA, USA). In the figures, asterisks denote *p* < 0.05 versus the relevant control group as specified in each figure legend.

## Results

### Effect of LED irradiation on pro-inflammatory cytokine expression in gingival fibroblasts

To screen the three wavelengths (525, 630, and 850 nm) for anti-inflammatory activity in periodontal cells, each wavelength was applied to HGFs in either preventive or therapeutic mode. In the preventive stage, cells were irradiated 30 min before LPS stimulation; in the therapeutic stage, irradiation was given 30 min after LPS. The energy density was held constant at 10 J/cm² across the three wavelengths.

Among the wavelengths tested in the pre-irradiation conditions, 630 nm light produced the largest reduction in transcript levels of IL-6 and IL-8 (Fig 1A). Relative IL-6 expression in the no-LED LPS group was 23.6 ± 1.7, and 630 nm irradiation lowered this value to 16.5 ± 2.2 (p < 0.05). A parallel decrease was observed for IL-8, from 298.0 ± 19.4 in the no-LED group to 269.1 ± 12.0 after 630 nm irradiation. Green (525 nm) and near-infrared (850 nm) light produced smaller and statistically non-significant trends in the same direction. When light was applied after LPS (therapeutic mode), 630 nm again significantly reduced IL-6 (39.7 ± 2.8 versus 30.6 ± 7.4) and IL-8 (752.7 ± 50.4 versus 644.3 ± 72.8) compared with the no-LED group (Fig 1B). The mRNA-level findings were corroborated at the protein level by ELISA: secreted IL-6 in HGF supernatants was significantly lower after 630 nm irradiation in both pre- and post-LPS settings (Fig 1C). To rule out a contribution of cytotoxicity, viability was measured by MTT at 24 h after each treatment, and no wavelength caused a significant change in viability (S3A Fig). These data identify 630 nm as the most effective wavelength for suppressing the cytokine response of HGFs to LPS, and they suggest that the effect is operative whether the light is delivered before or after the inflammatory stimulus.

**Fig 1:**
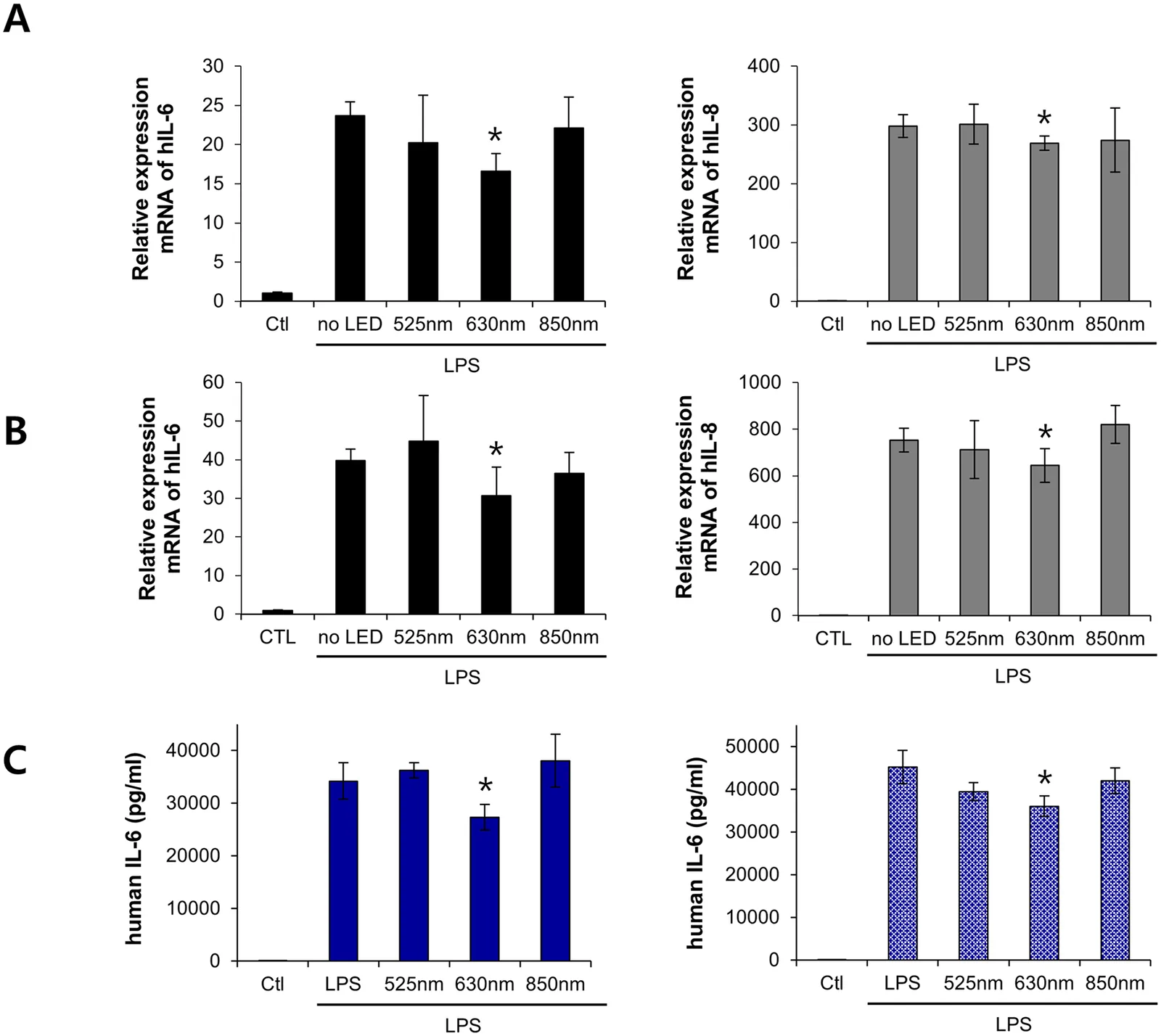
Effects of LED irradiation on the expression of pro-inflammatory cytokines in lipopolysaccharide (LPS)-stimulated human gingival fibroblasts (HGFs). (A) Relative mRNA expression levels of human IL-6 and IL-8 following pre-treatment with LED irradiation 30 min prior to LPS stimulation. (B) Relative mRNA expression levels of human IL-6 and IL-8 following LED irradiation applied 30 min after LPS stimulation. (C) Secreted IL-6 protein levels in HGFs culture supernatants quantified by ELISA, in the pre-irradiation (left) and post-irradiation (right) conditions. mRNA levels were normalized to GAPDH and are presented relative to the untreated control group. Data are expressed as mean ± SD from three independent experiments. *p < 0.05 versus the LPS-stimulated group without LED treatment (one-way ANOVA followed by Dunnett’s post hoc test).

### Effect of LED irradiation on pro-inflammatory cytokine and NO production in RAW 264.7 macrophages

The wavelength-dependent effects observed in HGFs were subsequently evaluated in RAW 264.7 macrophages, another major cell type involved in periodontal inflammation. RAW 264.7 cells were treated under the same pre- and post-irradiation schemes used for HGFs (10 J/cm²; 1 µg/mL LPS). In the preventive setting, both 525 nm and 630 nm significantly lowered IL-6, IL-8, and IL-1β transcript levels compared with the no-LED control, whereas 850 nm produced no significant change (Fig 2A). In the therapeutic setting, the same two wavelengths again reduced expression of all three cytokines, with 630 nm tending to produce the most consistent suppression across replicates (Fig 2B). At the protein level, secreted IL-6 measured by ELISA was lower in the 525 nm and 630 nm groups in both pre- and post-LPS conditions (Fig 2C, 2D). Viability of RAW 264.7 cells was unaffected by any wavelength tested (S3B Fig).

**Fig 2:**
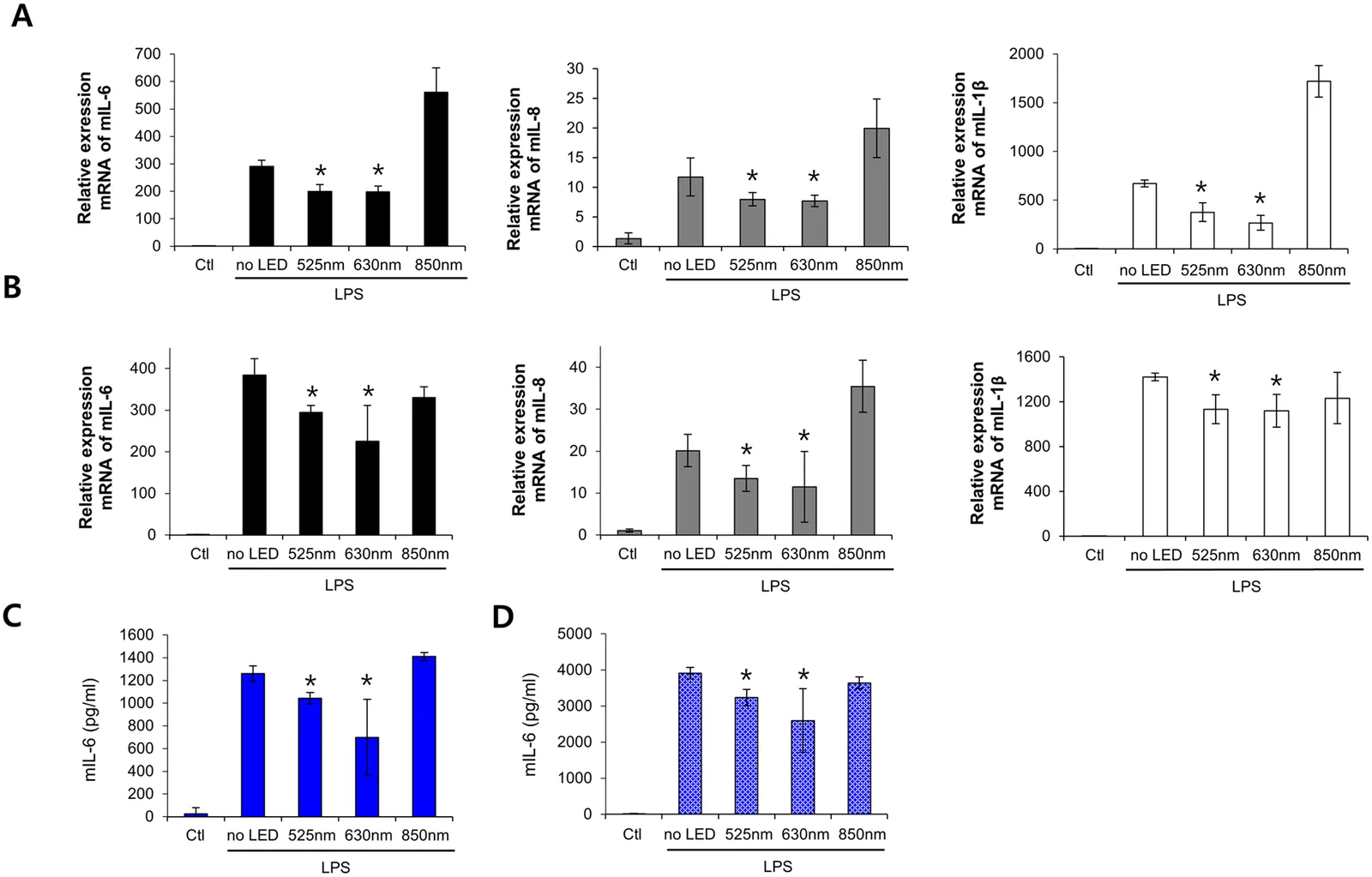
Effects of LED irradiation on pro-inflammatory cytokine expression and secretion in LPS-stimulated RAW 264.7 macrophages. (A) Relative mRNA expression levels of mouse IL-6, IL-8, and IL-1β following pre-irradiation with LED light prior to LPS stimulation. (B) Relative mRNA expression levels of the same cytokines following LED irradiation applied 30 min after LPS stimulation. (C, D) Secreted IL-6 in culture supernatants measured by ELISA in the pre- and post-irradiation conditions, respectively. mRNA values were normalized to GAPDH and are presented relative to the untreated control. Data are mean ± SD from three independent experiments. *p < 0.05 versus the LPS-stimulated no-LED group.

Nitric Oxide (NO) is a key effector molecule produced by activated macrophages and contributes to bystander tissue damage during periodontal inflammation. Nitrite accumulation was measured in the culture supernatants of LPS-stimulated RAW 264.7 cells with and without LED irradiation. In the preventive stage, 525 nm irradiation did not significantly alter nitrite levels, whereas 630 nm produced a clear reduction relative to the no-LED group (Fig 3A). In the therapeutic stage, both wavelengths significantly lowered nitrite (Fig 3B). The discrepancy between green and red light in the preventive setting suggests that NO regulation by PBM may depend on the timing of irradiation relative to the inflammatory stimulus, an observation addressed in the Discussion.

**Fig 3:**
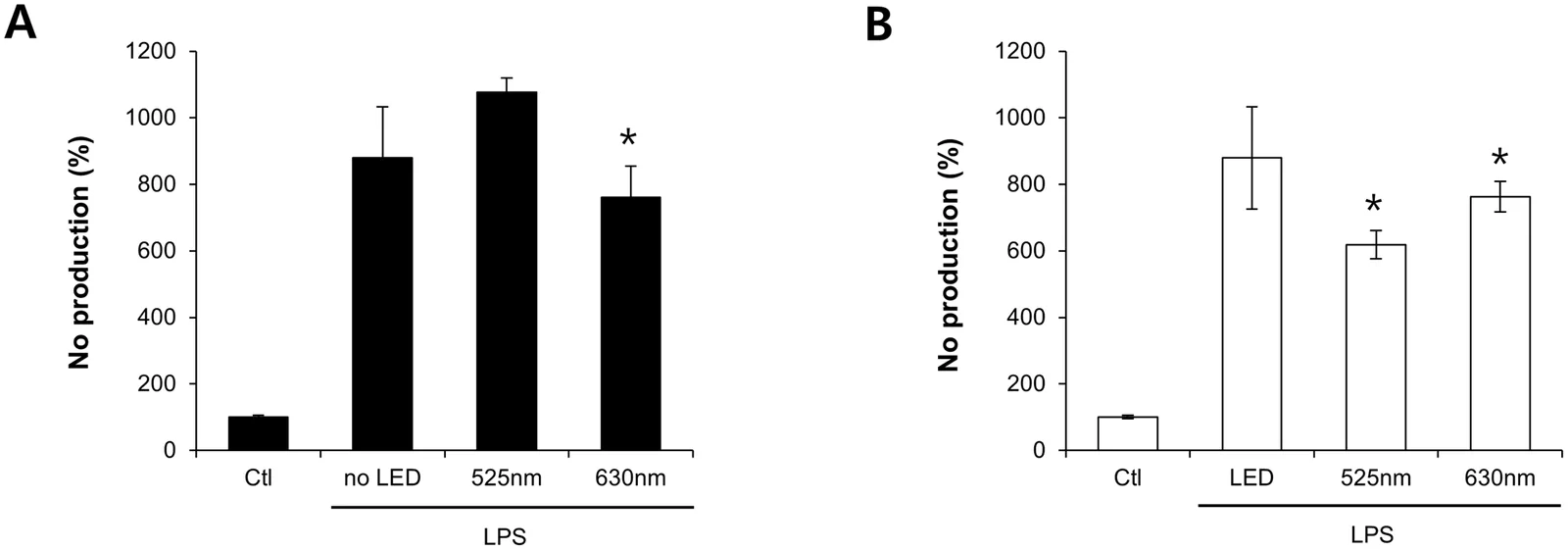
Effects of LED irradiation on nitric oxide (NO) production in LPS-stimulated RAW 264.7 macrophages. (A) Nitrite accumulation in culture supernatants when cells were pre-irradiated 30 min before LPS stimulation. (B) Nitrite accumulation when cells were irradiated 30 min after LPS stimulation. Bars represent mean ± SD from four independent experiments. *p < 0.05 versus the LPS-stimulated no-LED group.

### 630 nm LED irradiation enhances osteoblast differentiation

Having identified 630 nm as a wavelength that suppressed inflammatory responses in both HGFs and RAW 264.7 cells, the same wavelength was next examined for its effect on bone cell differentiation. The effects of 630 nm LED irradiation were first evaluated in osteoblasts because restoration of bone-forming activity is desirable in the context of periodontal therapy. Mouse primary calvarial osteoblasts were induced to differentiate with 100 ng/mL BMP2 and were irradiated at 0, 2.5, 5, or 10 J/cm² on days 0 and 4. On day 7, ALP staining and quantitative ALP activity assays were performed. Both staining intensity and enzymatic activity were significantly higher in cells irradiated at 5 and 10 J/cm² than in the non-irradiated control, whereas 2.5 J/cm² produced only a modest, non-significant trend (Fig 4A).

**Fig 4:**
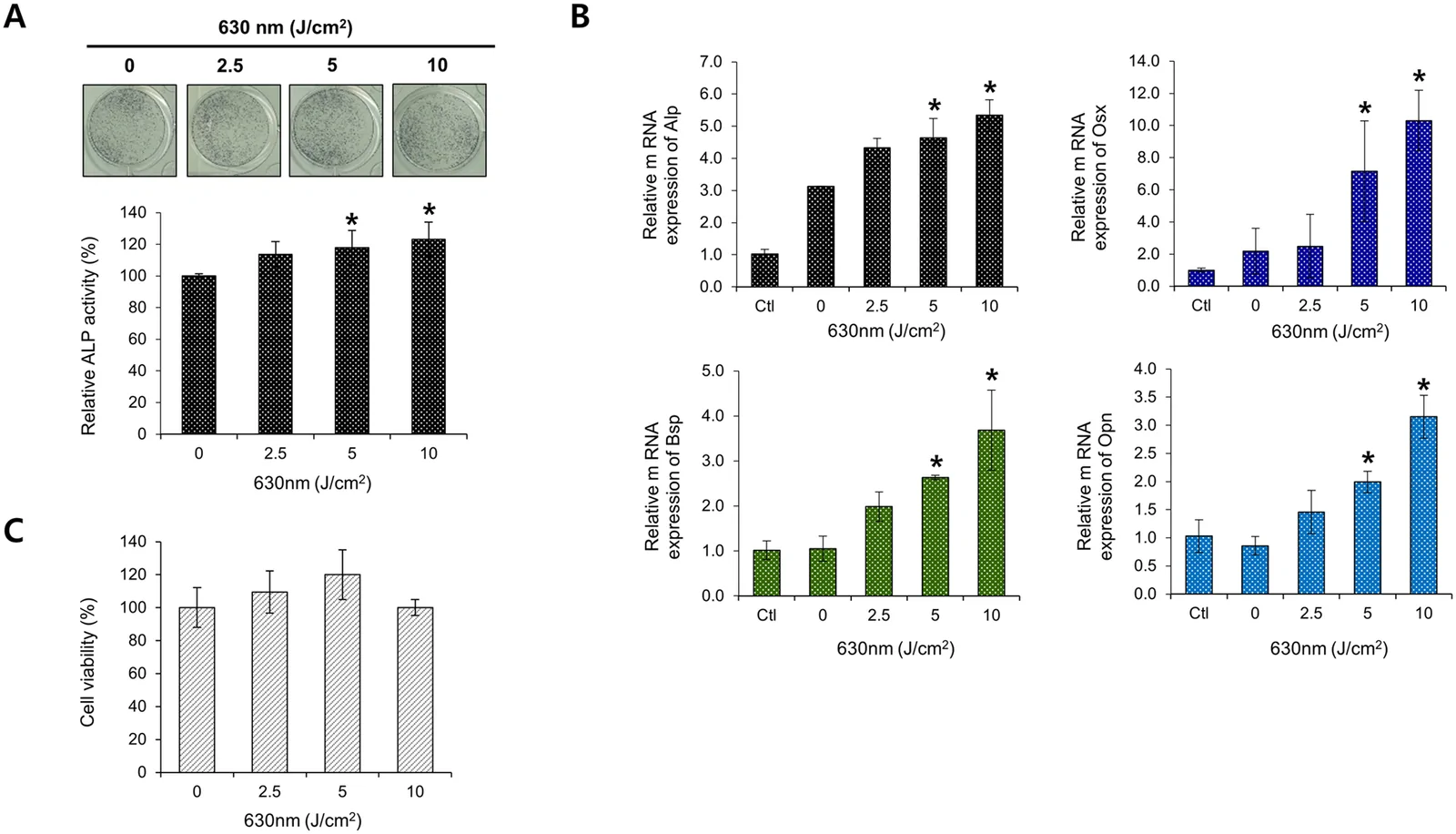
Effects of 630 nm LED irradiation on the osteogenic differentiation of mouse primary calvarial osteoblasts. (A) Representative images of alkaline phosphatase (ALP) staining (upper panel) and quantitative ALP enzymatic activity (lower panel) on day 7 of differentiation in cells irradiated at 0, 2.5, 5, and 10 J/cm². (B) Relative mRNA expression of osteogenic markers Alp, Osx, Bsp, and Opn measured by quantitative PCR. Gene expression was normalized to GAPDH and is presented relative to the non-irradiated control. (C) Cell viability assessed by MTT assay confirming that the irradiation conditions used were not cytotoxic. Data are mean ± SD from three independent experiments. *p < 0.05 versus the non-irradiated control group.

To complement the ALP readouts, the transcript levels of four canonical osteogenic markers — Alp, Osx, Bsp, and Opn — were measured by quantitative PCR. Each of the four genes was significantly upregulated by 630 nm irradiation at 5 and 10 J/cm² compared with control (Fig 4B). MTT analysis confirmed that the increases in osteogenic markers were not attributable to differences in cell number, since cell viability remained comparable across all irradiation conditions (Fig 4C).

To determine whether the osteogenic effect of 630 nm irradiation was mediated through canonical BMP signalling, Western blot analysis was performed on mouse primary calvarial osteoblasts cultured in BMP2-containing osteogenic medium for 7 and 14 days. RUNX2, the master transcription factor of osteoblast differentiation, was clearly induced in the 5 and 10 J/cm² groups relative to the non-irradiated control (Fig 5A). Phosphorylation of Smad1/5/9, the canonical downstream effector of BMP receptor signalling, was likewise increased at the same energy densities, while total Smad1 levels were unchanged. Densitometric quantification across three independent experiments confirmed these patterns (Fig 5B). These results suggest that 630 nm irradiation augments BMP/Smad-dependent osteogenic signalling and thereby promotes osteoblast maturation.

**Fig 5:**
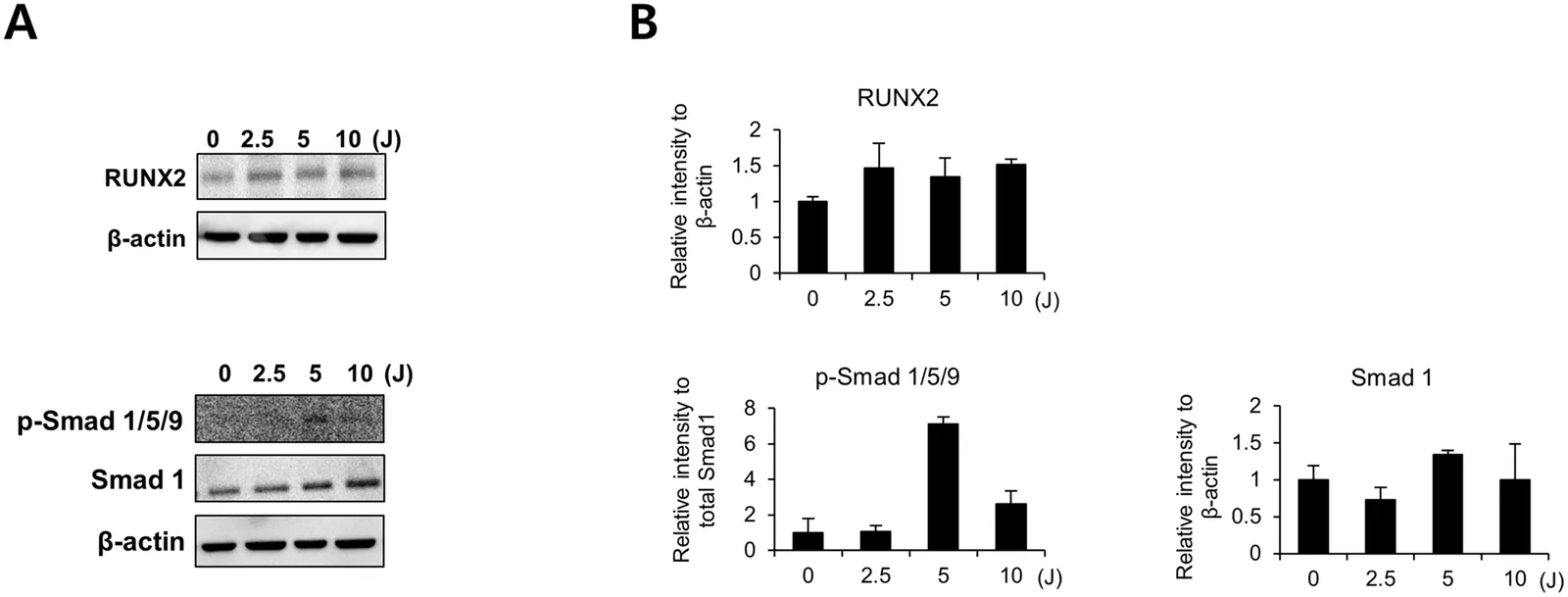
630 nm LED irradiation activates BMP-Smad-RUNX2 signalling in mouse primary calvarial osteoblasts. (A) Representative Wes (A) Representative Western blot panels showing RUNX2 protein (upper) and phosphorylated Smad1/5/9 together with total Smad1 (lower) in osteoblasts cultured in BMP2-containing osteogenic medium and irradiated at 0, 2.5, 5, and 10 J/cm². β-actin served as the loading control. (B) Densitometric quantification of RUNX2 (normalized to β-actin), phospho-Smad1/5/9 (normalized to total Smad1), and total Smad1 (normalized to β-actin) from three independent experiments. Band intensities were quantified using ImageJ. Data are mean ± SD. *p < 0.05 versus the non-irradiated control.

To verify the generality of this observation, the key experiments were repeated in the MC3T3-E1 pre-osteoblast cell line. Irradiation at 630 nm and energy densities of 5 and 10 J/cm² significantly increased ALP activity (S4A Fig). The same conditions upregulated Alp, Osx, Col1a1, Bsp, and Opn at the mRNA level (S4B Fig), and these effects were independent of cell viability (S4C Fig). The consistency of the response between primary calvarial osteoblasts and an established pre-osteoblast line strengthens the conclusion that 630 nm light, in the range of 5–10 J/cm², acts as a positive modulator of osteoblast differentiation.

### 630 nm LED irradiation does not affect osteoclast differentiation

Osteoclasts are the principal effectors of pathological bone loss in periodontitis, and an ideal phototherapy regimen would suppress, or at least not enhance, their differentiation. To examine the effect of 630 nm irradiation on osteoclastogenesis, BMMs were differentiated with M-CSF and RANKL, and were irradiated on days 0 and 2 at 0, 2.5, 5, or 10 J/cm². On day 4, TRAP staining was used to identify multinucleated osteoclasts. None of the irradiation conditions produced a significant change in the number of TRAP-positive multinucleated cells compared with the non-irradiated control (Fig 6A). Consistently, the expression of canonical osteoclast markers — c-Fos, NFATc1, and Ctsk — at days 1, 2, and 3 of differentiation showed no significant differences across irradiation conditions (Fig 6B).

**Fig 6:**
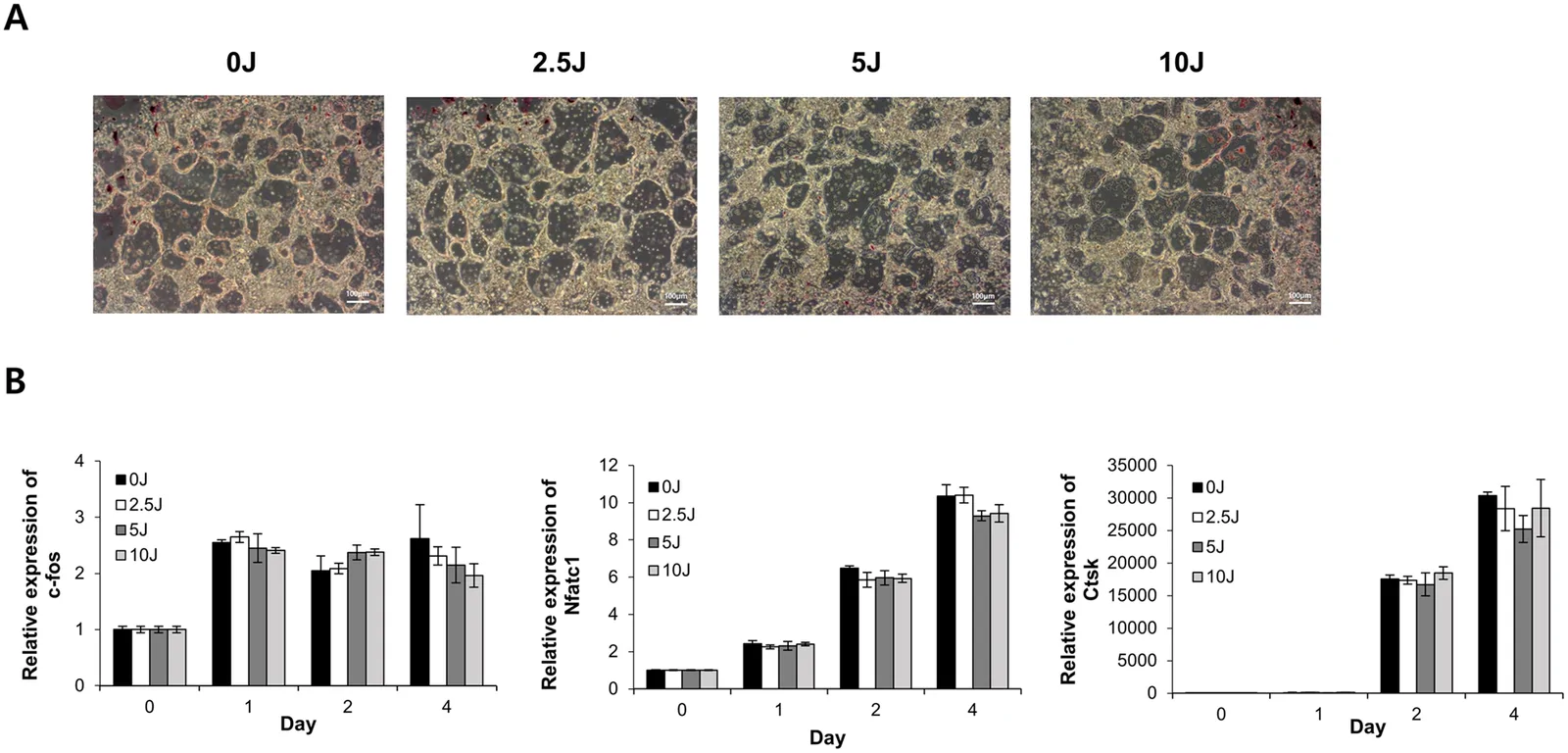
630 nm LED irradiation does not affect osteoclast differentiation of mouse bone marrow-derived macrophages (BMMs). BMMs were induced to differentiate with M-CSF (30 ng/mL) and RANKL (50 ng/mL), and were irradiated with 630 nm LED light on days 0 and 2 at the indicated energy densities. (A) Representative TRAP staining images on day 4 of differentiation. (B) Relative mRNA expression of osteoclast differentiation markers c-Fos, NFATc1, and Ctsk on days 0, 1, 2, and 4 of differentiation, normalized to GAPDH. Data are mean ± SD from three independent experiments. No significant differences were detected between irradiated and non-irradiated groups (one-way ANOVA).

The same experiments were repeated using RAW 264.7 cells as an alternative osteoclast precursor model. Again, neither TRAP-positive cell numbers nor the expression of osteoclast markers were significantly altered by 630 nm irradiation at any energy density tested (S5 Fig). Cell viability was preserved in both cell types under all conditions examined (S6 Fig). These results indicate that 630 nm LED irradiation, at the doses that promote osteoblast differentiation and suppress inflammation, is neutral with respect to osteoclastogenesis — neither inhibiting nor enhancing the formation of mature osteoclasts.

## Discussion

The aim of the current study was to evaluate whether LED irradiation at three wavelengths could modulate the two principal axes of periodontal pathology — soft-tissue inflammation and bone remodelling — within a single, internally consistent *in vitro* framework. The present study yielded three main observations regarding the effects of LED irradiation on inflammatory responses and bone-related cellular processes. First, among the wavelengths tested, 630 nm produced the most consistent reduction in pro-inflammatory cytokine and nitric oxide responses in both gingival fibroblasts and macrophages, and this effect was apparent whether the light was applied before or after LPS stimulation. Second, the same wavelength at 5 and 10 J/cm² promoted osteoblast differentiation in primary calvarial osteoblasts and in MC3T3-E1 cells, an effect that was accompanied by activation of RUNX2 expression and Smad1/5/9 phosphorylation. Third, 630 nm irradiation under the conditions tested did not enhance osteoclast differentiation in BMMs or RAW 264.7 cells, indicating a directional preference toward bone formation rather than resorption. Taken together, these findings support the idea that red-wavelength LED irradiation may exert combined anti-inflammatory and pro-osteogenic effects relevant to periodontal disease.

The anti-inflammatory response observed in HGFs is broadly consistent with earlier reports on red-light PBM. Previous studies showed that 635 nm LED irradiation reduced IL-6 and IL-8 expression in *Porphyromonas gingivalis* LPS-treated HGFs [16], while 630 nm light suppressed the inflammatory response of human periodontal ligament stem cells in part by enhancing intracellular ATP synthesis [19]. These findings extend the earlier observations in two practical directions. First, three wavelengths were directly compared within the same cellular system, which allows the relative efficacy of green, red, and near-infrared light to be compared under directly comparable experimental conditions. The more consistent effect of 630 nm over 850 nm in HGFs is somewhat unexpected, given that near-infrared light is generally believed to penetrate tissue more effectively. A plausible explanation is that the absorption peak of cytochrome c oxidase relevant to PBM responses lies closer to the red region, so that the efficiency of energy transfer to the photoacceptor outweighs the penetration advantage of 850 nm in a thin *in vitro* layer [10]. Second, the comparable efficacy of pre- and post-LPS irradiation suggests that 630 nm light could be applied prophylactically — for example, during periodontal maintenance — as well as therapeutically, after an inflammatory episode has begun. This dual mode of action distinguishes the present protocol from many earlier studies that examined only a single irradiation timing.

Reports on the effect of photobiomodulation (PBM) on nitric oxide (NO) production have been inconsistent. In lipopolysaccharide-stimulated RAW 264.7 macrophages, 780 nm diode laser irradiation increased nitric oxide production while reducing the expression and secretion of several inflammatory cytokines [33]. Similarly, 660 nm laser irradiation increased NO production under specific irradiation conditions while suppressing TNF-α and IL-6 [15]. These findings indicate that PBM may regulate NO and inflammatory cytokines differently depending on the wavelength and irradiation parameters. In the present study, 630 nm light reduced nitrite accumulation in both pre- and post-LPS settings, whereas 525 nm light reduced nitrite only when applied after the stimulus. One interpretation is that NO regulation by PBM is more sensitive to the timing of irradiation than cytokine regulation, presumably because NO production depends on the inducible isoform of nitric oxide synthase (iNOS) whose transcriptional induction is itself a dynamic and stimulus-dependent process. This argues for caution in extrapolating from a single irradiation protocol to general statements about PBM and NO.

The osteogenic findings deserve closer attention because they connect the anti-inflammatory effect of 630 nm light to a direct benefit for bone formation. The combination of increased ALP activity, upregulated transcript levels of multiple osteogenic markers, elevated RUNX2 protein, and enhanced phospho-Smad1/5/9 is consistent with a model in which 630 nm irradiation amplifies BMP receptor signalling. BMP2-induced Smad1/5/9 phosphorylation drives the expression of RUNX2 and Osx, which in turn transactivate downstream effectors such as Bsp, Opn, and Col1a1 [34]. The observation of consistent effects in both primary calvarial osteoblasts and MC3T3-E1 cells, and across two independent energy densities, argues against an artifact specific to one cell preparation. Mechanistically, the most plausible upstream event is the well-described action of red light on mitochondrial chromophores, which would increase ATP availability and shift the cellular redox state in a manner conducive to differentiation. Whether 630 nm light also influences alternative pathways — for example, β-catenin signalling, which is known to cooperate with BMP/Smad in osteoblast differentiation — remains to be tested.

Perhaps the most therapeutically relevant observation is that 630 nm irradiation did not promote osteoclast differentiation at any of the energy densities that promoted osteoblast differentiation. Reports on PBM and osteoclastogenesis have been mixed. Some authors have observed inhibition of RANKL-induced osteoclast formation by red light, while others have reported no effect or a modest enhancement, depending on the dose [35]. The present findings fall on the neutral side of this spectrum and, importantly, the lack of effect was reproducible across two independent osteoclast precursor systems. From a clinical standpoint, neutrality is in fact a favorable outcome: in the context of periodontal disease, where the bone remodelling balance is tilted toward resorption, a treatment that promotes bone formation without simultaneously stimulating resorption would be expected to restore homeostasis more effectively than one that affects both compartments.

Future studies should validate these findings in animal models of periodontitis and in inflammatory models using periodontal pathogens or polymicrobial biofilms. Further dose-response and mechanistic studies are required to determine the optimal irradiation conditions and clarify the roles of mitochondrial activity, nitric oxide signalling, and BMP/Smad/RUNX2 signalling. Long-term efficacy and safety should also be evaluated before clinical translation.

## Conclusions

Within the limits of an *in vitro* study, these findings suggest that 630 nm LED irradiation has a dual action that may be useful in periodontal therapy: it suppresses the inflammatory response of gingival fibroblasts and macrophages to bacterial LPS, and at the same time it promotes the differentiation of osteoblasts through activation of BMP/Smad/RUNX2 signalling, without enhancing osteoclast formation. The effect is operative whether the light is applied before or after the inflammatory stimulus, raising the possibility that the same protocol could be deployed in both prophylactic and therapeutic settings. Confirmation of these *in vitro* findings in animal models of periodontitis is a logical next step, and will determine whether 630 nm LED-based PBM can be developed as an adjunctive treatment for periodontal disease.

### Limitations of the study

Several limitations of the present work should be acknowledged. First, all experiments were conducted *in vitro* using either established cell lines or mouse primary cells; the translation of these findings to the periodontal tissue environment, where multiple cell types coexist and interact dynamically, remains to be established. Second, a single energy density (10 J/cm²) was examined for the inflammation experiments, which was chosen on the basis of preliminary range-finding but does not represent an exhaustive dose-response analysis. Third, the mechanistic claims regarding mitochondrial photoacceptors are inferred from the literature rather than measured directly; future work should include readouts such as mitochondrial membrane potential, cytochrome c oxidase activity, and intracellular ATP after irradiation. Fourth, the inflammatory stimulus used here was *E. coli* LPS, which is a well-validated TLR4 agonist but does not fully recapitulate the complex stimulus presented by oral biofilms. A confirmation of the present findings using *P. gingivalis* LPS or live bacterial challenge would strengthen the clinical relevance of the conclusions. Finally, the long-term effect of repeated LED irradiation on resident periodontal cell populations, and any potential off-target effects, will need to be addressed in animal models before the protocol can be considered for clinical translation.

## Supporting information

S1 Fig Custom-designed three-wavelength LED phototherapy device used in this study. (A–C) Emission photographs of the three wavelengths: (A) 525 nm (green), (B) 630 nm (red), and (C) 850 nm (near-infrared). Each panel shows the device in operation (upper) and the LED matrix surface (lower). (D) LED array layout (left) and the corresponding wiring diagram (right). Each LED measures 1.0 × 0.5 mm; 225 LEDs are arranged in a 15 × 15 matrix on a 5 × 5 cm board, giving a density of 9 LEDs/cm².
(TIF)

S2 Fig Schematic illustration and photographs of the experimental irradiation setup. (A) Three-dimensional schematic of the complete irradiation system, showing the DC power supply connected through banana sockets to the custom light-emitting diode (LED) module with an aluminium heat sink, mounted with a dark plate cover above the cell culture plate. (B) Representative photographs of the device in operation for each wavelength: 525 nm (green), 630 nm (red), and 850 nm (near-infrared), with the LED module connected to the power supply. (C) Cross-sectional schematic of the non-contact irradiation geometry, showing the LED module (525/630/850 nm source) with heat sink positioned above the culture plate, the direction of irradiation from above, and the fixed 1 cm working distance between the LED surface and the culture plate surface containing the culture medium. (D) Representative close-up photographs of a culture plate during irradiation with 525 nm (green), 630 nm (red), and 850 nm (near-infrared) light. Near-infrared (850 nm) light is not visible to the naked eye.
(TIF)

S3 Fig Cell viability of HGFs and RAW 264.7 macrophages following LED irradiation under inflammation experimental conditions. (A) Viability of HGFs assessed by MTT assay after LED irradiation applied either before (pre-irradiation) or after (post-irradiation) 1 µg/mL LPS exposure. (B) Viability of RAW 264.7 cells assessed under the same conditions. Data are mean ± SD from at least three independent experiments. No significant differences were detected.
(TIF)

S4 Fig Effects of 630 nm LED irradiation on the osteogenic differentiation of MC3T3-E1 pre-osteoblasts. (A) Representative ALP staining images (upper panel) and quantitative ALP activity (lower panel) at the indicated 630 nm energy densities. (B) Relative mRNA expression of osteogenic markers Alp, Osx, Col1a1, Bsp, and Opn measured by quantitative PCR and normalized to GAPDH. (C) Cell viability assessed by MTT assay, demonstrating that the observed pro-osteogenic effect was not attributable to changes in cell number. Data are mean ± SD from three independent experiments. *p < 0.05 versus the non-irradiated control.
(TIF)

S5 Fig 630 nm LED irradiation does not affect osteoclast differentiation of RAW 264.7 cells. RAW 264.7 cells were treated with 50 ng/mL RANKL to induce osteoclastogenesis and were irradiated with 630 nm LED light on days 0 and 2. (A) Representative TRAP staining images on day 3 of differentiation. (B) Relative mRNA expression of c-Fos, NFATc1, and Ctsk, normalized to GAPDH. Data are mean ± SD from three independent experiments. No significant effect of irradiation was detected.
(TIF)

S6 Fig Cell viability of BMMs and RAW 264.7 cells under 630 nm LED irradiation during osteoclast differentiation. Cell viability assessed by MTT assay confirmed that the irradiation conditions used in the osteoclast experiments (0, 2.5, 5, and 10 J/cm²) did not cause cytotoxicity in either BMMs (left) or RAW 264.7 cells (right). Data are mean ± SD from three independent experiments.
(TIF)

S7 Fig Original uncropped blot images and primary microscopy images. Uncropped images of all blots shown in Fig 5A, with the positions of the target bands indicated, and the original microscopy images corresponding to Fig 6A.
(PPTX)

S8 Table Individual replicate values underlying the bar graphs. Individual values for each bar graph in Figs 1A–1C, 2A–2D, 3A–3B, 4A–4C, 5B and 6B. Data are organized by figure panel.
(XLSX)
